# RADIOLOGICAL STUDY OF MEGACOLON IN *TRYPANOSOMA CRUZI* INFECTED RATS

**DOI:** 10.1590/0102-672020180001e1341

**Published:** 2018-03-01

**Authors:** Carlos Edmundo Rodrigues FONTES, Ana Paula de ABREU, Aretuza Zaupa GASPARIM

**Affiliations:** 1 Laboratory of Experimental Surgery; 2 Post-Graduate Program in Health Sciences, State University of Maringá, Maringá, PR, Brazil

**Keywords:** Chagas disease, Trypanosoma cruzi, Megacolon, Barium Enema., Doença de Chagas, Trypanosoma cruzi, Megacólon, Enema opaco.

## Abstract

**Background:**

Researches on Chagas disease still use several animals and rats, due to size and susceptibility were preferred by many authors**.**

**Aim:**

To develop an experimental model of megacolon in rats inoculated with the strain Y of Trypanosoma cruzi.

**Methods:**

Thirty male Wistar rats were distributed in three groups inoculated with different inoculants: Group A: 600000, Group B: 1000000 and Group C: 1500000 blood trypomastigotes of T. cruzi. Animals were sedated intramuscularly at zero inoculation time (T_0_) and 60 days after inoculation (T_60_), to perform the barium enema in order to evaluate the dilatation of the different segments of colon in a comparative study of the measurements obtained, using a digital caliper. Evidence of infection was performed by blood smear collected from the animal’s tail 18 days after inoculation with observation of blood forms.

**Results:**

Comparing the intestinal diameter of the inoculated animals with 60,0000 trypomastigotes in the T_0_ of infection with T_60_ days after the inoculation, significant dilatation was observed between the proximal, medial and distal segments (p<0.01), indicating the establishment of the megacolon model. In addition, comparing intestinal diameter between the different segments, with in the T_0_ of infection and the T_60_ after inoculation, significant alterations were observed (p<0.05).

**Conclusion:**

The proposed model was possible for in vivo studies of alterations due to infection by T. cruzi and functional alterations of the colon. In addition, the changes manifested in the colon are not directly proportional to the size of the inoculum, but to the time of infection that the animals were submitted, since the animals inoculated with 60,0000 blood forms were the ones which presented the most significant alterations.

## RESUMO

**Racional:**

Pesquisas para doença de Chagas ainda utilizam diversos animais e o rato por seu tamanho e sua suscetibilidade foi o preferido por muitos pesquisadores.

**Objetivo:**

Desenvolver um modelo experimental de megacólon em ratos inoculados com a cepa Y de Trypanosoma cruzi.

**Métodos:**

Utilizou-se 30 ratos, machos, distribuídos em três grupos inoculados com diferentes inóculos: Grupo A: 600000, Grupo B: 1000000 e Grupo C: 1500000 tripomastigotas sanguíneos da cepa Y de T. cruzi. Os animais foram sedados via intramuscular no tempo zero de inoculação (T_0_) e aos 60 dias após a inoculação (T_60_) para realização de enema opaco para avaliação da dilatação dos diferentes segmentos do cólon em estudo comparativo das medidas obtidas, com o auxílio de um paquímetro digital. A comprovação da infecção foi realizada com esfregaço de sangue coletado a partir da cauda do animal 18 dias após a inoculação com observação das formas sanguíneas.

**Resultados:**

Ao comparar o diâmetro intestinal dos animais inoculados com 60.0000 formas tripomastigotas no T_0_ de infecção com T_60_ dias após a inoculação, observou-se dilatação significativa entre os segmentos proximal, medial e distal (p<0,01), indicando o estabelecimento do modelo de megacólon. Além disso, ao comparar o diâmetro intestinal entre os diferentes segmentos, dentro do T_0_ de infecção e do T_60_ após a inoculação, observou-se alterações significantes (p<0,05).

**Conclusões:**

O modelo proposto mostrou-se factível para estudos in vivo das alterações decorrentes da infecção pelo T. cruzi e alterações funcionais do cólon. Além disso, as alterações manifestadas no cólon não são diretamente proporcionais ao tamanho do inóculo, mas sim ao tempo de infecção que os animais foram submetidos, visto que os inoculados com 600000 formas sanguíneas foram as que mais apresentaram alterações significantes.

## INTRODUCTION

Chagas disease (CD) or American trypanosomiasis is an important anthropozoonosis that has as etiological agent, the protozoan hemoflagellate *Trypanosoma cruzi,* as vector hematophagous insects of the order Hemiptera, family Reduviidae and subfamily Triatominae^3^ ^,^ ^5^. It is estimated that around eight million people are infected worldwide, occurring more frequently in Latin America, where it is endemic^25^.

In Brazil, the indeterminate or asymptomatic form is the most common (60-70%), followed by cardiac and digestive ones (20-30% and 8-10%)^1^. However, in Central Brazil and Chile, the digestive tract of CD is predominant, but it is not reported in Venezuela and Central America^8^ ^,^ ^6^. These differences may be associated to several factors, such as the genetic lineage of the parasite, geographic distribution and the patient’s immune status^9^.

The clinical manifestations of CD can be classified in the acute and chronic phase, acute phase with high parasitemia being able to be symptomatic or asymptomatic and duration of approximately two months^16^. The chronic phase begins with predominance of the indeterminate form, in which it is characterized by a long latent period and low parasitemia, which can last for 10-30 years. After this period many infected patients may present compromised organs such as heart, esophagus and colon, characterizing cardiac and digestive forms^16^.

The digestive form is characterized by megaesophagus and/or megacolon resulting from inflammation and fibrosis of the esophagus and/or colon, leading to destruction of the autonomic nervous system and organ dysfunction^11^. Megacolon is caused by myenteric plexular denervation in the intestinal mucosa, causing motility disorders associated with colonic constipation and dilatation^4^ ^,^ ^20^. Such complications can often be fatal resulting in perforation of the intestine and toxic megacolon and death^18^.

Studies on CD have been conducted since its discovery. Therefore, several experimental models have been used^12^ ^,^ ^14^ ^,^ ^15^. In the first study developed by Oswaldo Cruz, monkeys were used as experimental models, *Callithrix Penicillata* was inoculated with isolated parasites sent by Carlos Chagas, and trypomastigotes were found in the blood 30 days after inoculation^10^ ^,^ ^13^ ^,^ ^22^ ^,^ ^24^. *Cebus* monkey was used by Torres&Tavares (1958)^24^ for research on myocarditis^17^, and *Rhesus* monkey by Guimarães and Miranda to study the megaesophagus^10^. 

Researches for CD also used many other animals, such as the guinea pig for organ study; dogs for the size to study heart lesions; mice for the size and susceptibility were preferred by many researchers^7^ ^,^ ^12^.

Taking in consideration such context, it is necessary to develop and implement a therapeutic proposal for experimental models through laboratory animals.

 This study aimed to develop an experimental model of megacolon in rats inoculated with the *T. cruzi* Y strain to prove the development of the digestive form of the disease.

## METHODS

This study was approved by the Committee of Ethical Conduct on the Use of Animals in Experimentation of the State University of Maringá under (n^o^ 046/2009) following the ethical principles in animal experimentation, adopted by the Brazilian Society of Science in Laboratory Animals.

### Parasite

Strain Y (TcII)^19^ ^,^ ^26^ ^,27^ was used from the acute phase patient, which was cryopreserved in liquid nitrogen in the trypanosomatids collection of the University’s CD laboratory.

### Animals and inoculum

Thirty male Wistar rats (*Rattus novegicus albinus*), aged eight weeks and weighing between 180-200 g were used. The animals were harvested from polypropylene cages coated with dry sawdust in ideal conditions of temperature (20-25°C), humidity (70%), light-dark cycle, with water (chlorinated) and ration (Nuvilab Cr-1® from Nuvital®) available at will. The animals were divided into three groups containing 10 animals each, inoculation was subcutaneously with different concentrations of the parasite: group A: 600000 blood trypomastigotes/0.1 ml of blood; group B: 1000000/0.1 ml blood; and group C: 1500000/0.1 ml of blood^2^.

### Barium enema

Animals were sedated by administration of ketamine hydrochloride and xylazine hydrochloride in 1:1 ratio given intramuscularly. After sedation, were placed on their own table. For the performance of the barium enema, a catheter of nelaton n^o^18, rectally was given and 5 ml of barium sulfate administered with time of controlled administration and radiographed. The evaluation of the dilation of proximal, medial and distal colon segments with a focus distance of 1.5 m and exposure time of 1 s in radiological equipment of 50000 Ma with the aid of a digital caliper was used to determine the time zero (T_0_). 

After radiological examination the animals of groups A, B and C were inoculated as previously described. Eighteen days after inoculation, 5 μl of blood was collected for examination^2^. Sixty days after inoculation (T_60_) animals were sedated and the enema was repeated to evaluate the dilatation of the different segments of the colon.

Comparisons were made between the measurements obtained at T_0_ and T_60_.

### Determination of infectivity rates

Infectivity rate was obtained by the ratio between the number of infected animals and the number of animals submitted to the X100 test. Animals presenting at least one blood form per field were considered infected.

### Statistical analysis

Obtained data was entered in a spread sheet of the Microsoft Excel 2010 program and analyzed statistically with the aid of BioEstat 5.0^®^. Distribution of the data was verified with the Shapiro-Wilk test. Since data presented normal distribution, it was expressed as mean±standard deviation. ANOVA-Tukey test was used to compare the groups. The level of significance adopted in the tests was 5%, so associations with p<0.05 were considered significant.

## RESULTS

The animals of groups A, B and C were submitted to examination 18 days after inoculation, and it was possible to determine the infectivity rate of 100%. Group A animals inoculated with 600000 blood trypomastigotes / 0.1 ml blood had 11.5 mm intestinal diameter at the T_0_. In the T_60_, significant dilatation of 50.4% (p<0.01) was observed in the proximal colon, 36.1% (p<0.05) in the medium colon and 47.6% (p<0.05) in the distal colon, respectively (Table 1).

**TABLE 1:** Mean diameter and standard deviation of the proximal, middle and distal segments of the colon of Wistar* rats inoculated with different inoculum: 600000 (A), 1000000 (B) and 1500000 (C) trypomastigotes of the *T. cruzi* Y strain.

Mean diameter of the colon segment (mm)							
		T_0_			T _60_		
Group	Inoculum	Proximal	Middle	Distal	Proximal	Middle	Distal
A	600000	11,5^a^±1,6	7,0^a^ ± 0,9	6,6^a^ ± 0,8	17,3^b^±1,8	9,5^b^±1,1	9,7^b^±1,4
B	1000000	12,6±1,3	11,2±2,5	8,9^a^±1,91	16,8±2,3	11,0±1,9	10,5^b^±1,3
C	1500000	15,0±1,4	10,1±1,2	10,2^a^±1,3	18,9±1,8	11,5±1,2	11,7^b^±1,7

*At time zero (T_0_) and 60 days (T_60_). Different letters on the same line represent significant differences (p<0.01)

**FIGURE 1:**
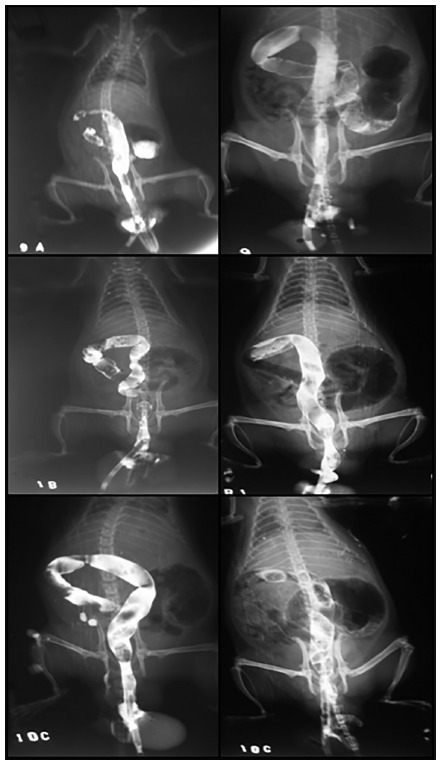
Radiological examination (barium enema) of groups A, B and C (T_0_) on the left and A B and C (T_60_) on the right, showing dilation of the proximal and distal colon. Group A presents double contrast image in small intestine due to incompetence of the ileocecal valve, and group C fecal image in the colon showing difficulty in colonic emptying.

In groups B (1000000 blood trypomastigotes/0.1 ml blood) and C (150000 blood trypomastigotes/0.1 ml blood) changes were observed only in the distal colon with dilatation of 18.7% and 15.0% (p<0.01, Table 1, Figure 1), respectively. Comparing the different colon segments at T_0_ and T_60_ for group B and C, significant changes were observed (p<0.05, Table 1, Figure 1).

It is possible to observe that the appearance of the megacolon is not directly related to the quantity of the inoculum, but to the time of infection to which animals were submitted. Comparing the intestinal diameter of the animals inoculated with 600000 trypomastigotes forms at T_0_ infection at 60 days post- infection, significant dilatation was observed among the proximal, medial and distal segments (p<0.01), indicating that the rat Wistar was a good experimental model for megacolon studies. In addition, comparing the intestinal diameter between the different segments within T_0_ and T_60_, a variation was observed between them (p<0.05).

## DISCUSSION

In the present study Wistar rats were used to develop an experimental model for the digestive alterations caused by CD. Wistar rats were chosen for being easy to handle, as well as for the protocols for their use, such as anesthesia and care. 

However, another important point to be analyzed is the animal’s age; younger ones were the most susceptible to disease^7^ ^,^ ^17^. Only males were used for this experimental model to avoid alterations related to the hormonal cycle of females^22^. According to Soares et al. (2012)^2^, studying females mice infected with *T. cruzi* showed to be more resistant to disease than males.

Although literature demonstrates that the amount of the inoculum is directly proportional to the severity of the disease^10^ ^,^ ^13^ ^,^ ^22^ ^,^ ^24^, such results showed that the observed changes in the colon diameter of the animals are not directly related to the inoculum size, but to the time of infection to which animals were submitted.

According to experiments obtained at the university laboratory, there is a great difference in resistance mechanisms between mice and rats infected by *T. cruzi*. Mice inoculated with 1400 blood trypomastigotes in 0.1 ml of Y strain blood exhibits fur and neurological changes. 

Although the inoculums used in rats in this study was larger than the used in mice, no clinical alterations were observed in the studied groups during the 60 days period. 

The performance of the barium enema was able to evaluate the existence or no existence of dilatation of colon, characterizing the intestinal form of the CD that results from dilatation, elongation and hypertrophy of the wall of colon, as consequence of the injuries of the musculature and the neurons of the enteric nervous system, especially the Meissner and Auerbach plexuses. This dilation was present in all groups evaluated at higher or lower rates; however, in group A the greatest alterations were observed.

There was difficulty in administering the contrast due to the presence of accumulated stool, result of the complete emptying of colon in the examination after 60 days, which caused some animals to have reflux of the barium by the anus. This fact can be explained by dyskinesia of the colon by the disease, and it was not observed in animals at T_0_ (before inoculation). Such difficulty was also reported by Okumura, (1961)^14^. The use of a digital caliper was able to precise measurement of the organ diameter.

During the exams, distension of the abdomen could be observed as contrast was injected. After 60 days (groups B and C), progression of air was observed in the radiographs of group C. In addition, double contrast formation with dilation of the cecum and small intestine occurred, demonstrating incontinence of the ileocecal valve, a result similar to the one described by Okumura& Correia (1953)^13^ in mice.

## CONCLUSION

The proposed model proved to be feasible for in vivo studies of alterations caused by *T. cruzi* infection and functional alterations of the colon. In addition, the changes manifested in the colon are not directly proportional to the size of the inoculum, but to the period of time of infection to which animals were submitted; those inoculated with 600000 blood forms showed the most significant alterations. The results demonstrated the importance of continuing the use of the rats to study the functional alterations of the colon.
